# A Deformable Smart Skin for Continuous Sensing Based on Electrical Impedance Tomography

**DOI:** 10.3390/s16111928

**Published:** 2016-11-16

**Authors:** Francesco Visentin, Paolo Fiorini, Kenji Suzuki

**Affiliations:** 1 Department of Intelligent Interaction Technologies, University of Tsukuba, Tsukuba 305-8573, Japan; 2 Department of Computer Science, University of Verona, 37057 Verona, Italy; paolo.fiorini@univr.it; 3 Faculty of Engineering, Information and Systems, University of Tsukuba, Tsukuba 305-8573, Japan; kenji@ieee.org

**Keywords:** deformable, adaptable, wearable sensor, electrical impedance tomography

## Abstract

In this paper, we present a low-cost, adaptable, and flexible pressure sensor that can be applied as a smart skin over both stiff and deformable media. The sensor can be easily adapted for use in applications related to the fields of robotics, rehabilitation, or costumer electronic devices. In order to remove most of the stiff components that block the flexibility of the sensor, we based the sensing capability on the use of a tomographic technique known as Electrical Impedance Tomography. The technique allows the internal structure of the domain under study to be inferred by reconstructing its conductivity map. By applying the technique to a material that changes its resistivity according to applied forces, it is possible to identify these changes and then localise the area where the force was applied. We tested the system when applied to flat and curved surfaces. For all configurations, we evaluate the artificial skin capabilities to detect forces applied over a single point, over multiple points, and changes in the underlying geometry. The results are all promising, and open the way for the application of such sensors in different robotic contexts where deformability is the key point.

## 1. Introduction

Robots and robotic devices are continuously improving and speeding up steps in our life, by becoming more ubiquitous and present both in the workplace, and in other daily activities. As the integration progress is becoming more popular, additional precautions have to be taken into account in order to ensure safety for the humans and objects that surround the robot. The common approach that separates humans and robots using protective cages is no longer adequate, especially when future scenarios where a close collaboration between the robot and its human operator are considered. Consequently, it is important to provide solutions that implement sensing capabilities to robots in order to collect information about the surroundings and thus plan safemotion-paths that avoid static and moving objects and—in case of imminent collision—minimise the damages by performing an avoidance manoeuvre or gently stopping its action. Besides safety, the interaction itself is another important aspect that should be taken into account. Tactile and haptic feedback from humans can be a useful additional and more direct channel of communication that can result in a more natural and understandable interaction method. In fact, information provided by touch is important for humans, since from those it is possible to extract valuable data that can help to understand the meaning of the interaction itself. Having such capabilities will help in the development of the next generations of robots capable to interact not only with humans but also with other robots in a smoother way.

The field of artificial sensing is very promising; for this reason, in the last 30 years [1], many researchers made it the focus of their work. Particular interest has been placed on the problem of grasping control with robotic hands [2]. With the growth of interest in humanoid and companion robots, many researchers started to investigate on large scale robotic sensing [3] in order to provide full-body scale sensing to these machines. Even if many solutions have been developed in the last years, tactile sensing is a very challenging problem, and it involves several engineering issues that often require special processing equipment. In addition, there are fundamental technological difficulties that limit the direct transition from the development of a single working sensor to a more complex network of sensors that is spread over large surfaces. However, different solutions that tackle and try to overcome the problem can be found in the literature. These arrays of sensors—commonly referred to as *artificial skins*—consist of a group of distributed sensors that are connected together in order to provide information over the sensing area. The sensor network is usually considered large (i.e., able to completely cover a wide portion of the robotic device) and able to conform over different 3D shapes—including curved and mobile parts—for which it was designed.

In order to replicate the capabilities of human skin, a diversity of sensing principles have been adopted in the development of the single sensing elements composing an artificial skin [4]. These, used alone or in combination with others, allowed the development of artificial skins capable of distinguishing between different phenomena, such as pressure, vibration, and temperature. Regarding tactile sensing, the main transduction methods identified so far belong to the following classes: resistive, capacitive, piezoelectric, magnetic, optical, and ultrasonic. Among these, capacitive sensing has gained importance, especially in consumer electronics. The technology has excellent sensitivity, good spatial resolution, and large dynamic range. Capacitive sensors are very susceptible to noise, however (especially over large surfaces), they suffer from stray capacitance, and the measurement system can become complicated as the size of the sensor grows. Similarly, resistive sensors (e.g., piezo-resistive, and strain gauges) are among the most-used technology; their success is due to their low cost, and their high spatial and temporal sensitivity. The weakness of the technique lie in the low repeatability rate, high hysteresis (especially for piezo-resistive sensors), and noise sensitivity. All of these weaknesses can limit the application of resistivity-based sensors. Ultrasonic sensing—i.e., using resonant properties as a means of detecting changes over the domain—has the major advantage of using simple circuitry: a vibration speaker and a piezo-electric microphone. The technique provides developers with the possibilitiy of flexible design, and does not use conductive or magnetic components. This makes it safe for direct interaction and it is non-susceptible to electromagnetic interference. However, even if used in combination with a material that efficiently conducts vibration, it is dumped in function with the distance from the source. Thus, if the artificial skin is large, the sensitive area is limited to a small region between the speaker and microphone. Other limitations are related to the material, shape, and ambient noise. Optoelectronics are also a good candidate for pressure sensors. The technique allows the development of (semi-)transparent devices that are fast in response, have good sensing capabilities with good repeatability and reliability, and are immune to electromagnetic fields. However, the need of large external hardware makes this technology difficult to be used.

An additional shared disadvantage among the presented transducing mechanisms is the need for wires to connect the different sensing units that are part of the network. The use of a large number of distributed wires not only forms an excellent antenna for electromagnetic noise, but also reduces the mobility of the robot by making it more bulky in order to contain all the components. This issue is especially crucial in the field of soft robotics [5,6], where robots not only move more naturally by mimicking biological systems, but are also made of material (e.g., silicon rubber) that can deform and easily adapt to different situations. These novel technologies have more demands than more traditional robotic platforms, since it is fundamental to maintain their *soft* properties in order to be advantageous. The use of this paradigm will open a new path for the development of novel robotic systems that are intrinsically compliant and safer in case of accidental collision of the robot with a human. Sensors (or artificial skins) for these types of robots should be flexible and able to follow the changes undergoing in the robot structure, while maintaining sensing capabilities—even under large deformations.

Among the various approaches that can be used in such contexts, the one we found the most promising is the use of a tomographic technique known as Electrical Impedance Tomography. In this technique, a small current is injected into a conductive domain, and electric potentials are then measured around the boundaries in order to infer the internal structure of the domain. Approaches that use this technique are not new, and they have been used in different application domains, spanning from geophysical inspection [7,8] to biomedical measurements [9,10], and recently, robotic applications [11,12]. In all of these applications, Electrical Impedance Tomography has been used to localise areas of inhomogeneity within the domain caused by the presence of elements that change the domain conductivity. If applied to a stretchable conductive substrate (as in [13,14,15,16,17]), the technique can be used to develop a flexible sensor with arbitrary size and shape that does not suffer from the presence of wires within the sensing area. Even if it sounds promising, the technique has some drawbacks, especially related to spatial resolution.

By exploiting this sensing technique, the aim of this paper is to investigate and present a low-cost wearable sensor that is able to adapt to different surfaces and still provide information, even when deformed. This sensor can be promising in the field of soft robotics where current solutions cannot be used for scalability issue and the presence of stiff components. Considering this research domain and all the related problems, our contribution is the development of a sensor, in the form of a smart skin that can adapt its shape to the underlying structure and return information even when both the skin and the structure change shape. Compared to the current EIT-based smart skins, the sensor differ in the material used for its development and the application field. Our sensor is small in size, so the circuitry and the cable assemble that compose the whole electronics do not interfere in the morphological changes of the device where it is embedded, and modular in order to adapt to different situations.

We tested our system in different scenarios, and it proved to be as effective as the state of the art in this field. The paper is structured as follows: in Section 2, the technology used and the working principles that lay beneath it are described. Section 3 presents the experimental setup and protocol used to gather and analyse the data from each experiment. Section 4 and Section 5 present the results and the discussion according to what has been found. The last Section is left for the conclusions and future works.

## 2. Material and Methods

### 2.1. Electrical Impedance Tomography

Electrical Impedance Tomography (EIT) [18] is an established imaging technique used to reconstruct the internal structure of a domain by estimating its conductivity distribution, derived as a result of applying different patterns of electrical current and measurements of electric potentials. EIT was initially introduced to retrieve the internal admittance of a conductive body in [19,20], respectively, in the field of medical and geophysical exploration. Its first clinical use was related to the identification of the different tissues inside a human chest. This was made possible by using an array of 100 electrodes placed on one side of the chest, and a single electrode earthed on the other side. Shortly after, the first clinical impedance tomography system was developed in the Department of Medical Physics in Sheffield, UK. The system has been used to perform different clinical studies, and it is still in use in many centres today for imaging lung ventilation, cardiac function, gastric emptying, brain function and pathology, and screening for breast cancer. EIT imaging has not only been used in clinical applications; from its early stages, it was also being used in geophysical analysis, and more recently in industrial applications (e.g., pipe inspection, or fracture detection in structures), and in robotics.

In a typical EIT application, one or more arrays of electrodes are equally placed at the boundaries of a conductive body. At every step of the measurement process, among these electrodes, two are selected to be used to inject electrical current into the domain (driving electrodes), while all the others (measurement electrodes) are used to acquire the electric potentials—measured with respect to a common ground—which is generated at the boundaries of the domain. According to the geometry of the studied domain, number and position of the electrodes it is possible to define specific injection/measuring patterns in order to reconstruct the internal conductivity of the studied domain.

From a mathematical point of view, EIT consists of solving a forward and an associated inverse problem [21]. In the forward case, the electric potentials at the boundaries of the considered domain are estimated by assuming a known uniform conductivity of the domain and the value of the injected current. A common approach to dealing with these pieces of information is to describe the real shape of the domain using Finite Element Analysis (FEM), in which the continuous form of the current propagation problem is transformed into a discrete approximation. The estimated values are then compared with the measured ones to update the model with the correct conductivity value. The updated model is then used as reference to solve the associated inverse problem. Solving such a problem is not straightforward, since it is non-linear and ill-posed [22]. Common approaches to overcoming this kind of numerical issue consist of adding prior information about the domain to the model, using regularisation over the data in order to obtain a nearly well-posed problem that can be solved, and implementing an iterative algorithm that can provide an approximate solution.

As mentioned earlier, the use of EIT has several potential advantages when compared to other tomographic techniques. Most importantly, the technique itself can be considered safe in many applications, since it is non-invasive, and does not use ionising radiation. Furthermore, most of the developed systems are non-cumbersome (making them highly portable) and of reasonable manufacturing cost. However, there are also potential disadvantages. Among these, the most prominent is the relatively low spatial resolution which precludes the use of the technique to obtain precise morphological information as opposed to other tomographic techniques. Spatial resolution depends on various parameters and can be improved by increasing the number of electrodes [23,24] used with the drawback of an increase of the acquisition time. Another technical issue is associated with the fixed placement of electrodes; this is crucial in media that can be deformed or that can change their shape.

### 2.2. Reconstruction Process

A key component in the reconstruction process consists of the selection of the most appropriate sequence of injection and measurement patterns. These are strongly dependent on the application, noise level in the measurements, the geometry of the domain, and the electrode placement. A pattern consists of sequences of numbers that—at every step of the acquisition process—identify which of the electrodes act as the current source, the current sink, or are used to measure the electric potential. As a general practice, a pattern is designed to use every single electrode in at least one of its possible configurations.

**Figure 1:**
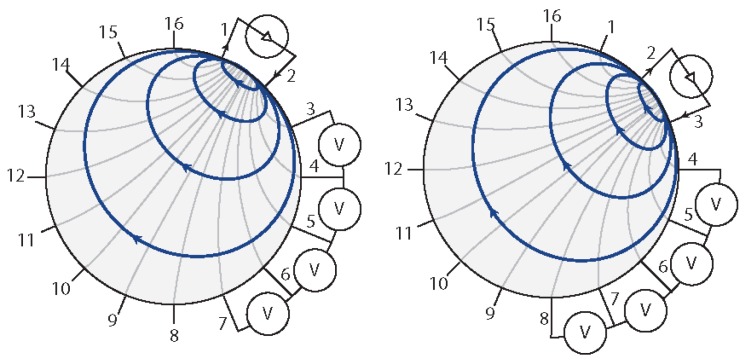
Example of bipolar adjacent pattern. Two electrodes are selected as the driving ones, while all the others are used to measure the electric potentials generated between adjacent pairs. The injection and measurement pattern is than switched by changing the driving electrodes until a full cycle is completed. Electric potentials measured at the driving electrodes are commonly not taken in order to ensure stability in the acquired data. In the image, dark-grey lines indicate iso-potential areas, while the current flow is shown in blue.

It is possible to classify the most-used patterns according to the number of current sources that are used in the injection part: *bipolar patterns* [18] use only one source, while *optimal patterns* [25,26] use multiple independent sources. The classification can be further refined by considering the relative position of the electrodes or the relationship between the electrical currents that are injected. Despite their name, optimal patterns are not the most-used ones. In fact, such patterns require a careful pre-tuning of the system, since the injected current cannot be obtained analytically. In addition, they are less tolerant to noise, to the electrode position and their contact impedance, and to the correct modelling of the forward problem. These patterns have the potential to produce more accurate images than the ones returned by the bipolar pattern. Bipolar patterns, on the contrary, are less prone to modelling error; thus, it is possible to use them in different situations. This peculiar property—in addition to the hardware simplification brought by the use of a single current source—makes bipolar patters the most used for EIT applications. Among these, *adjacent* [27] patterns are the most popular and easy to implement. These patterns use a pair of adjoining electrodes, both in the current injection and for the electric potential measurements. An example of a typical measurement sequence is depicted in Figure 1.

Once a full cycle of measurements has been performed, the electric potentials are then processed by an inverse problem solver algorithm to reconstruct the internal structure of the studied domain. An initial practical method used for this purpose was a non-iterative linear method known as back-projection [28]. The method is fast and is commonly used in various tomographic imaging techniques, such as X-ray scans. By the use of this approach, the equipotential volume between a pair of electrodes is back-projected along the whole boundary of the body. By combining all the projections, it is possible to estimate the position of the different conductive regions within the domain. While back-projection is conceptually simple, it does not correctly solve the EIT problem, since the current does not move in a straight line (i.e., differently from X-rays), but floods a region from source to drain. An example is given by the iso-potential lines shown in Figure 1. As a consequence, the reconstructed image results are blurred with some clear artefacts. In addition, when higher dimensions than 2D are considered, the technique suffers from additional limitations. For this reason, different novel approaches have been implemented. These consist of the use of deterministic algorithms based on the computation of the Jacobian—the linearised mapping between the boundary electric potential and the internal conductivity—of the discrete forward solution. The use of such algorithms allows for more precise solutions, even for higher-dimensional domains. Two practical approaches to reconstructing the inhomogeneity map are: (i) using static imaging; (ii) or using dynamic imaging. In static imaging, the absolute value of the conductivity distribution is reconstructed using an iterative approach. This method is extremely sensitive to uncertainty in electrode position, and it is generally slow, since at every step of the iteration, the FEM model has to be updated with the acquired measured data. The dynamic imaging approach, on the contrary, uses non-iterative one-step algorithms to reconstruct the differences in the conductivities between the two time frames. This is done by comparing a reference measurement—where homogeneous conductivity all over the domain is assumed—with later ones. Since the comparison is performed only on the changes in electric potentials, the method is fast, and it allows possible problems due to unknown contact impedance between electrodes and the measured media or problems related to incorrect positioning of the electrodes to be overcome. In both approaches, it is common practice to pre-condition the system using a regularisation method. This allows movement from an ill-posed problem to a similar well-posed one by attenuating the distribution of the null values in the matrices that are the cause of numerical instability.

Initial tests for the reconstruction process were performed using an open-source Matlab toolbox—Electrical Impedance Tomography and Diffuse Optical Tomography Reconstruction Software (EIDORS [29]). In order to provide more direct control of the parameters of the inverse solver algorithm, and to interface it with the acquisition system, we designed and implemented a modified version of the Matlab toolbox.

### 2.3. Electronics

According to the type of pattern an EIT system can handle, these are designed to implement one or more electrical current generators to create time-harmonic Alternating Current (AC) signals that are further modulated and tuned according to the application domain and the studied media. Although it is also possible to inject voltage and measure current, electric potential measurement is a more common practice; this is mostly because of the effects of the contact impedance between the electrodes and the conductive domain. These effects are significantly reduced by the large input impedance characteristics of voltmeters as compared to the low input impedance of ammeters. In addition, the hardware required to precisely measure the generated current requires high-cost components that have to be finely tuned in order to provide reliable measurements. As an alternative to AC currents, it is possible to use Direct Current (DC). The use of the latter is more convenient, since it allows to simplify the hardware required to both generate the electrical current and to read-out the measurements. In addition, the analysis result is simplified, since only the resistive component, and not the permittivity, is measured.

**Figure 2:**
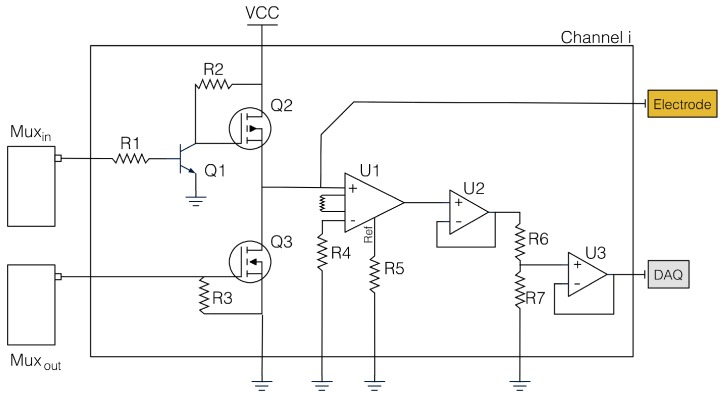
Schematic overview of the driving and readout electronics. The image depicts the configuration for a generic channel and its components. By using this configuration, a single channel can be set as: (i) current source; (ii) current sink (ground electrode); or (iii) used to measure the electric potentials with respect to an external common ground. The process is controlled by the two independent de-multiplexers connected at each channel. DAQ: data acquisition board. In the figure, Q1 is the NPN transistor, Q2 and Q3 respectively the P-MOSFET and N-MOSTEF, U1 is the INA118, U2 is the OPA827, and U3 is the TLV2371.

Starting from these considerations, we designed a simplified version of a commercial EIT system to be used as main component of our sensing unit. The main focus was placed on developing a low-cost adaptable system that is able to perform measurements by applying different patterns, and which is powered by a DC source. The use of DC current simplifies the hardware requirements, and allows the use of the developed hardware in battery-driven applications. The electronic system is composed of two main components printed on two separate printable circuit boards (PCBs): a voltage-to-current generator, and a driving/read-out circuit. Figure 2 shows a schematic representation of the driving and readout electronics for a given channel.

The current generator is implemented by a voltage regulator (Texas Instruments LM317 ) set up as precision current-limiter circuit. The output current value is controlled by a variable resistor, and it can be adjusted linearly by changing the resistor value. In order to provide a constant current supply on varying load, a 12 V voltage source is used as the input to the voltage regulator. The electrical current is then multiplexed across the different channels by the driving/read-out electronics.

Each channel in the driving/read-out electronics is designed to operate at three independent stages. To provide such capability, we implemented it as a half H-bridge, where a n-MOSFET (International Rectifier IRLML6344TR) and a p-MOSFET (International Rectifier IRLML9301TR) act as two independent switches connected to the same path. In this configuration, the p-MOSFET is connected to the positive source, and the n-MOSFET to the ground. By opportunely turning on and off the single switch, it is possible to set each channel to act as current source, current sink, or setting it to its high-impedance state, in which the channel can be used to measure the voltage. As initial choice, the control of the driving/read-out electronics was performed directly by the microcontroller. In order to reduce the number of cables and add modularity to the system, we moved to an integrated solution that uses two independent de-multiplexers (Texas Instruments CD74HC237M). To capture the smaller voltage variation, we added an amplification stage to each channel. It consists of a low-power general-purpose instrumentation amplifier (Texas Instruments INA118) with variable gain. The output of this initial stage is referred to as the ground. The signal then passes trough two subsequent op-amp (respectively, Texas Instruments OPA827 and TLV2371), both configured in a non-inverting gain configuration.

The driving/read-out electronics used in this paper has eight independent channels. However, the system has been designed to be modular to easily adapt its use to different requirements. Each channel is connected to the object under study by a single-ended cable that has an alligator clip or a with push button at its extremity. The acquired signal is routed toward an analogue pin of the micro-controller (Atmel ATmega2560). Since each channel is measured independently, it is possible to perform any possible pattern combinations either at run-time or in post-processing. It is worth mentioning that in the current version of the acquisition system, no further processing of the signal (e.g., noise filtering or signal conditioning) has been performed.

### 2.4. Sensor Fabrication

**Figure 3:**
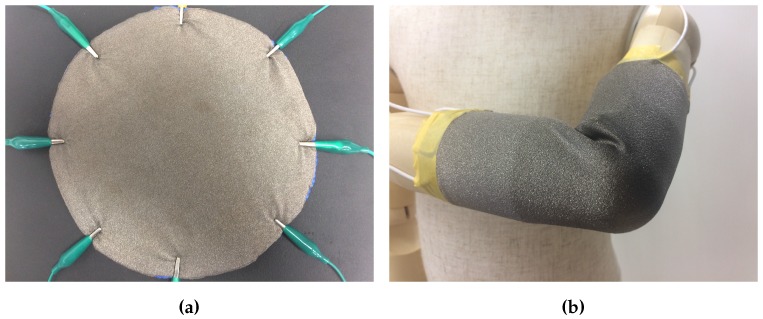
Smart skin with wiring. (**a**) The conductive textile is attached over a stretchable foam that allows better discrimination between normal forces and stretching, and creates an insulation layer that allows the artificial skin to be applied over different materials. The current version of the system uses only eight channels that are connected by alligator clip to the measurement system. (**b**) Possible application scenario attached over a dummy doll arm.

To exploit EIT as a tomographic technique, it is fundamental that the media over which the measurements are taken is conductive—best results are achieved when the conductivity is homogenous over the whole domain. In order to let our sensor achieve flexibility and adaptivity to various geometries, we used a highly conductive medical grade textile (Statex, Shieldex MedTex180) as sensing material. The material changes its conductivity non-linearly when stretched along any of the two directions. The conductive layer was firmly attached to a thin, stretchable foam substrate. This sightly limits the stretchability of the whole sensor, but allows the conversion of normal forces (i.e., vertical forces, such as touch) into noticeable local in-plane deformations. An additional benefit inherited from the use of the substrate consists of the possibility of applying the artificial skin over different materials while maintaining its insulation from the injected electrical current. The sensing area of the artificial skin has a circular shape, with a diameter of 200 mm. Figure 3 shows an overview of the artificial skin in two different application scenarios.

## 3. Experimental Evaluation

In this section, we will present the experimental setup used to characterise the behaviour of the sensor under different scenarios. In the first phase, electric potentials are acquired both in the absence and in the presence of different loads applied over the artificial skin. These initial measurements are fundamental to the creation of a series of reference values for the conductivity reconstruction. In the second phase, we applied the reconstruction algorithm to prove the capability of the system to correctly identify the presence of the probe and its position. In the last phase, more complex interactions–such as multi-touch identification and application over curved surfaces–were addressed to prove the artificial skin’s capabilities.

### 3.1. Experimental Setup

**Figure 4:**
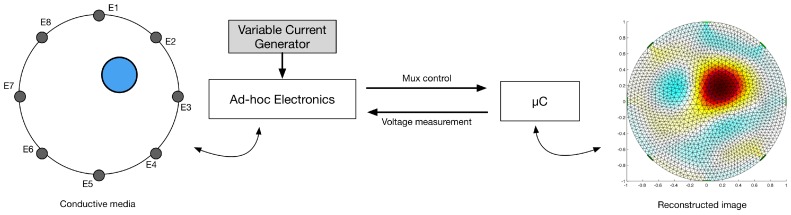
Schematic view of the experimental setup. The artificial skin is connected by wire to the ad-hoc developed driving/read-out circuit. This is connected to a microcontroller that sets the status of the different channel and functions as a digital acquisition board. The whole system is then connected to a laptop on which the main program and the inverse solver are running.

The experimental setup employed for the characterisation of the smart skin system consists of the components schematically illustrated in Figure 4. The artificial skin is connected to the driving/readout electronic previously described. This is controlled by an external ATmega2560 powered micro-controller that is used to both switch the status of the different channels and as data acquisition board (DAQ). A laptop with Matlab installed is connected to the micro-controller and used to control the whole system, and to perform the inverse problem solving.

To prove the adaptability of the system to react to and detect various stimuli, we used different probes that vary in size and shape. For instance, we used three different circular probes with radii ranging from 15 mm to 25 mm, and a square head probe with a size of 15 mm. In all of the experiments, in order to cope with possible fluctuations of the acquired value, a series of ten measurements were acquired, and then the mean value was used. In all of the experiments (unless stated otherwise), the artificial skin was placed on a flat surface.

For the reconstruction process, we used a dynamic imaging approach. A reference measurement was acquired and stored to be compared with later ones. Although the EIT community is moving toward a unified approach to reconstruct 2D medical images [30], as inverse solving algorithm we used the Gauss-Newton EIT reconstruction approaches, which have been widely used in EIT since the late 1980s [31]. The approach allows to represent the solution as a linear reconstruction matrix, which can be pre-computed and thus allows rapid, real-time imaging. The parameter used for the inverse solver were derived empirically by measuring the error of the reconstruction using different probes and then by selecting the best solution. In order to provide consistency in the reconstruction, the parameter values remain the same during all the experiments, unless otherwise stated.

### 3.2. Experimental Protocol

Different tests were performed to assess the overall performance of the sensors, according to the following protocols.

#### 3.2.1. Preliminary Analysis on the Sensor Working Principle

A first series of experiments was performed in order to capture the output value of each electrode in the absence of any force applied over the sensing area. At first, the output of an arbitrarily-chosen electrode was directly monitored and acquired with Matlab during a full driving cycle. In order to obtain valid measurements, we waited a fixed amount of time of 2μs (empirically calculated) to ensure electromagnetic stability before acquiring the data. The obtained value can be considered as the offset for the electric potential measurements to be used in later phases, where differences in the measured data correspond to the presence of inhomogeneity within the sensing layer due to external stimuli (i.e., touch or stretch).

#### 3.2.2. Indentation Tests and Electrode Sensibility

Following the results obtained in the first series of experiments, we continued by evaluating the changes in conductivity as a function of size and position of different loads applied over the sensing area. In order to better understand this phenomenon, we applied the probe in the region directly facing each electrode. The tests were performed using different probes with different sizes and shapes while keeping the applied force constant. For each configuration, we acquired the data 10 times and then averaged them to obtain a more stable waveform.

#### 3.2.3. Pressure Map Reconstruction

The artificial skin functionality was validated by means of indentation tests with the different probes as indenter. The measures obtained in the previous experiments were then used to tune the inverse problem solver, thus reconstructing the conductivity map associated with the artificial skin. The series of experiments was aimed at proving the capability of the inverse solver to detect the position and size of the different probes used in the tests. Once the reconstruction was performed, a peak detection was first done to identify the position and the area associated to the inhomogeneity, and then a value thresholding was done according to the peaks’ value. In this phase, we only consider single events that occur over the surface.

#### 3.2.4. Multi-Pressure Test

To prove the capability of the artificial skin to detect multiple contacts at the same time, the system was placed on a flat surface, and different contacts were achieved on its sensing area by means of hand-held probes with a circular section. Each load was added sequentially, and then removed in the same order. During the experiment, we did not consider the nominal force applied on each probe. Additional testing to improve object discrimination capabilities and pressure measurements will be left as future work.

#### 3.2.5. Application over Curved and Deformable Surfaces

With the last series of experiments, we wanted to prove the adaptability of the artificial skin to different substrates—in particular, to curved and deformable ones. In order to proceed in this direction, we first placed the artificial skin over different cylindrical surfaces with different radii. Afterwards, we placed the sensor over other objects, such as a bendable mannequin arm, the back of a chair, and around a deformable foam-made cylinder. Where the underlaying object did not provide sufficient support for the artificial skin, we placed the object over a table. Once the artificial skin was applied over these geometries, we performed similar experiments as in the case of the flat surface ones. During these test, we focused or attention on: (i) the capability of the system to detect a change of shape; and (ii) the robustness of the sensing technique under large deformations. In the first case, we used as reference voltage the ones taken on a flat surface, and we compared those with the one taken when the sensor was wrapped around the curved surface. In the second case, instead, both the reference and later measurements were taken directly when the artificial skin was placed over the curved surface.

## 4. Results

The results of each single experiment are reported in the following sections, subdivided by their respective categories.

### 4.1. Working Principle and Material Characterisation

**Figure 5:**
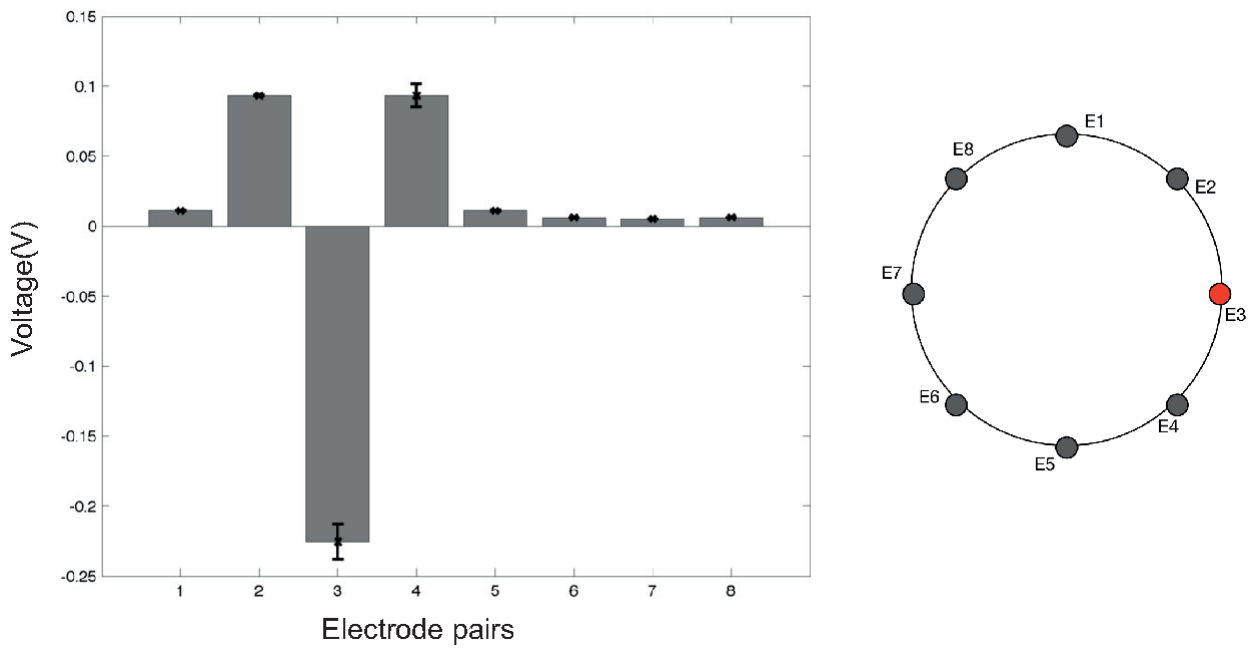
Electric potentials measured at Electrode 3 (E3) in an eight-channel configuration. Measurements were taken using an *adjacent pattern*, in both excitation and acquisition phases. The positive peaks correspond when the electrode is “close” to ground nodes. On the contrary, the negative peak occurs when E3 is used as a current sink. Error bars show the variability of measurements calculated over 10 samples.

**Figure 6:**
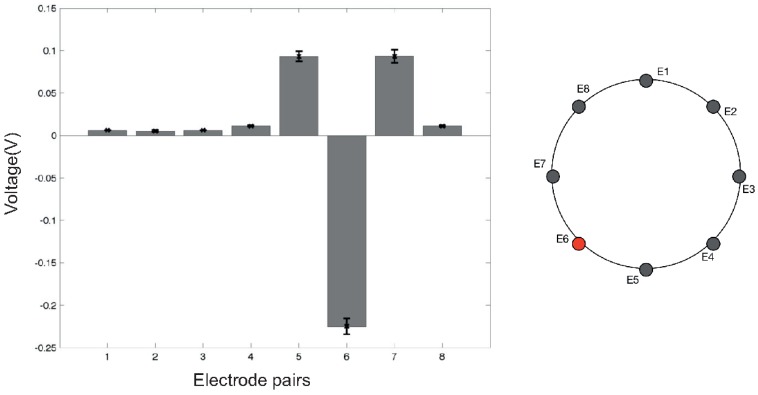
Electric potentials measured at Electrode 6 (E6) in an eight-channel configuration. Measurements were taken using an *adjacent pattern* in both excitation and acquisition phases. The positive peaks correspond when the electrode is “close” to ground nodes. On the contrary, the negative peak occurs when E6 is used as a current sink. Error bars show the variability of measurements calculated over 10 samples.

**Figure 7:**
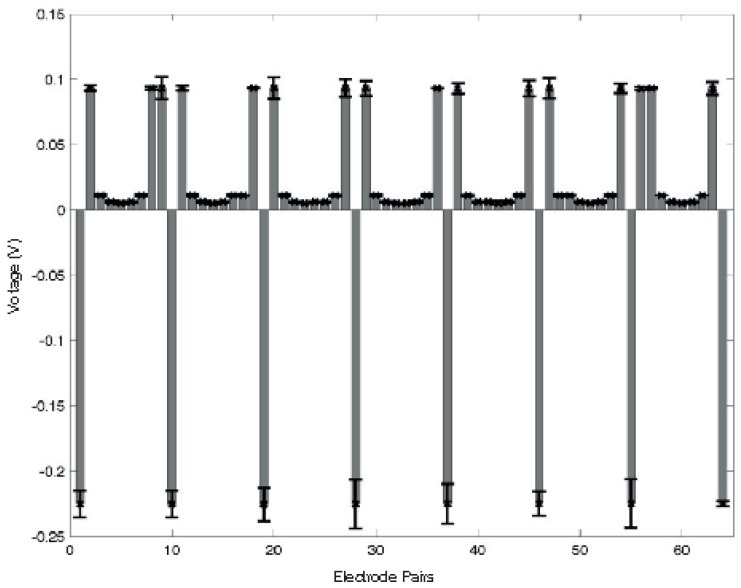
Electric potentials measured at each electrode pair by using an adjacent pattern in both excitation and acquisition phases. The measurements were taken when no load was applied over the artificial skin.

Initial tests have been performed in order to understand the behaviour of the conductive material—used as sensing layer—when an electrical current flows through it. Due to the high conductivity of the material (<1Ω/sq), 100 mA of current was required in order to achieve acceptable electric potential levels at the boundary. In a future version of the sensor, we are planning to move to a more resistive material in order to reduce the electrical current required for the system. During the characterisation process, no physical contact or force was applied over the sensing area of the smart artificial skin. Figure 5 and Figure 6 show the electric potential measurements at Electrodes 3 and 6, respectively, during a full cycle of excitations. The values were acquired using adjacent pattern; thus, each value in the graph represents the potential calculated between two adjoining electrodes. As the measurements suggest, the maximum and minimum variations can be found when the driving electrodes are close to the one where the measurements were taken. The assembly of all the measurements acquired from all the electrodes represents a reference voltage, $V_{0}$, used to calculate differences in the electric potentials when an event occurs over the sensor surface. Figure 7 shows an example of the reference measures.

### 4.2. Single Point Contact—Signal Indentation

**Figure 8:**
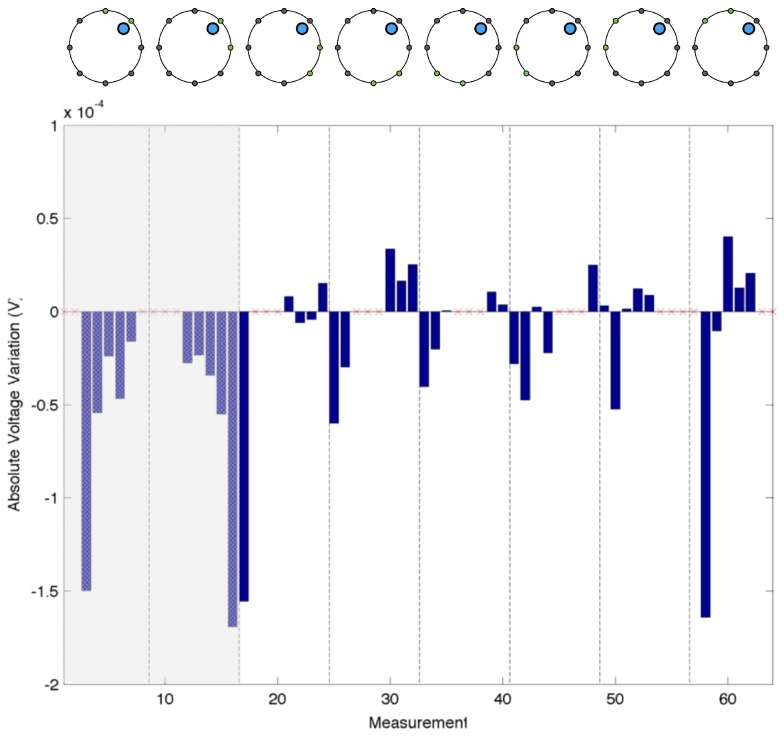
Electric potential measurements acquired when a probe is acting in the region facing Electrode 2 (E2). The values are grouped according to which electrode is used as ground following an adjacent pattern (highlighted in the top part of the figure). The shaded portion of the graph corresponds to the configuration when E2 is one of the driving electrodes. The major noticeable differences are the ones wherein E2 is involved in the measurements.

**Figure 9:**
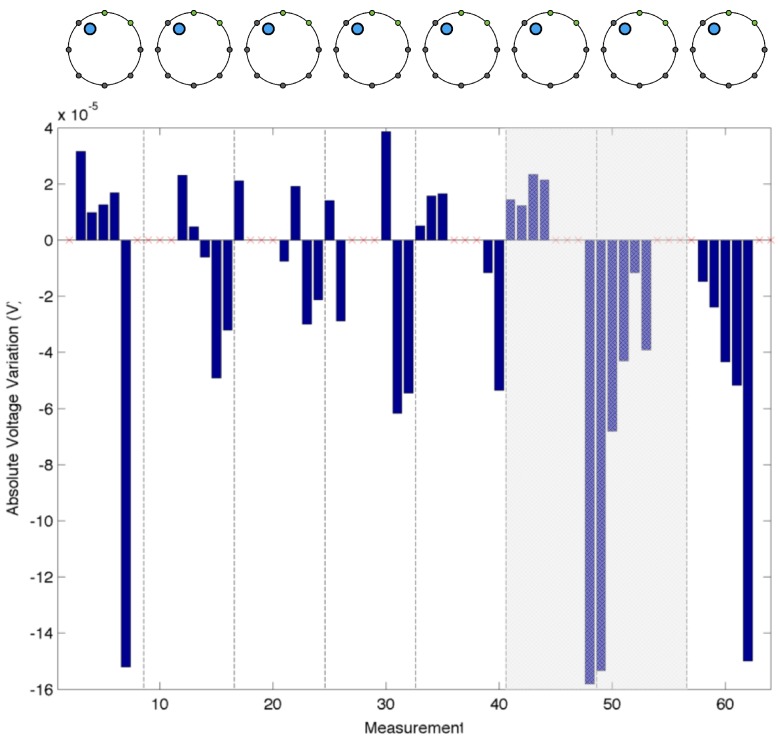
Electric potential measurements acquired when a probe is acting in the region facing Electrode 7 (E7). The values are grouped according to which electrode is used as ground following an adjacent pattern (highlighted in the top part of the figure). Not considering the region where E7 is acting as driving electrode (shaded area), the major noticeable differences are the ones wherein E7 is involved in the measurements (i.e., the last two bars in each group).

Using the same electrode configuration as in the previous experiments, we evaluate the changes in the acquired electric potentials as a consequence of an applied load over the sensing area. To better notice the phenomena, we chose the position of the probe as 5–10 mm apart from the electrode under test. For this specific test, we used only one probe with a circular section (15 mm radius), over which we applied a constant force of 3 N. By simply comparing the measurements in the presence and absence of the applied load, it is not possible to clearly appreciate the difference between them. A more clear understanding is noticeable by considering the absolute variation ($V_{meas}-V_{0}$) between the two measurements. Figure 8 shows the absolute difference using the value acquired by Electrode 2 (E2) when the probe was paced in the region facing the electrode. In the figure, the different measurements are grouped according to the electrode acting as ground. Each bar represents a voltage difference computed over the adjacent electrode; e.g., the first bar refers to electrodes E1–E2, the second to E2–E3, and so on, until all the electrode pairs are considered. Shaded regions are not to be considered since the electrode under consideration was acting as current source or connected to ground. In the figure, major negative changes occur in the electrode pairs that involve E2 (i.e., the first three bars in each group). The changes are a consequence of the presence of the probe that, acting on the conductive surface, changes the local conductivity of the layer. It is possible to notice a similar pattern when any of the other electrodes are considered. As reference, Figure 9 shows the absolute electric potential when a load was acting in the region facing Electrode 7 (E7).

**Figure 10:**
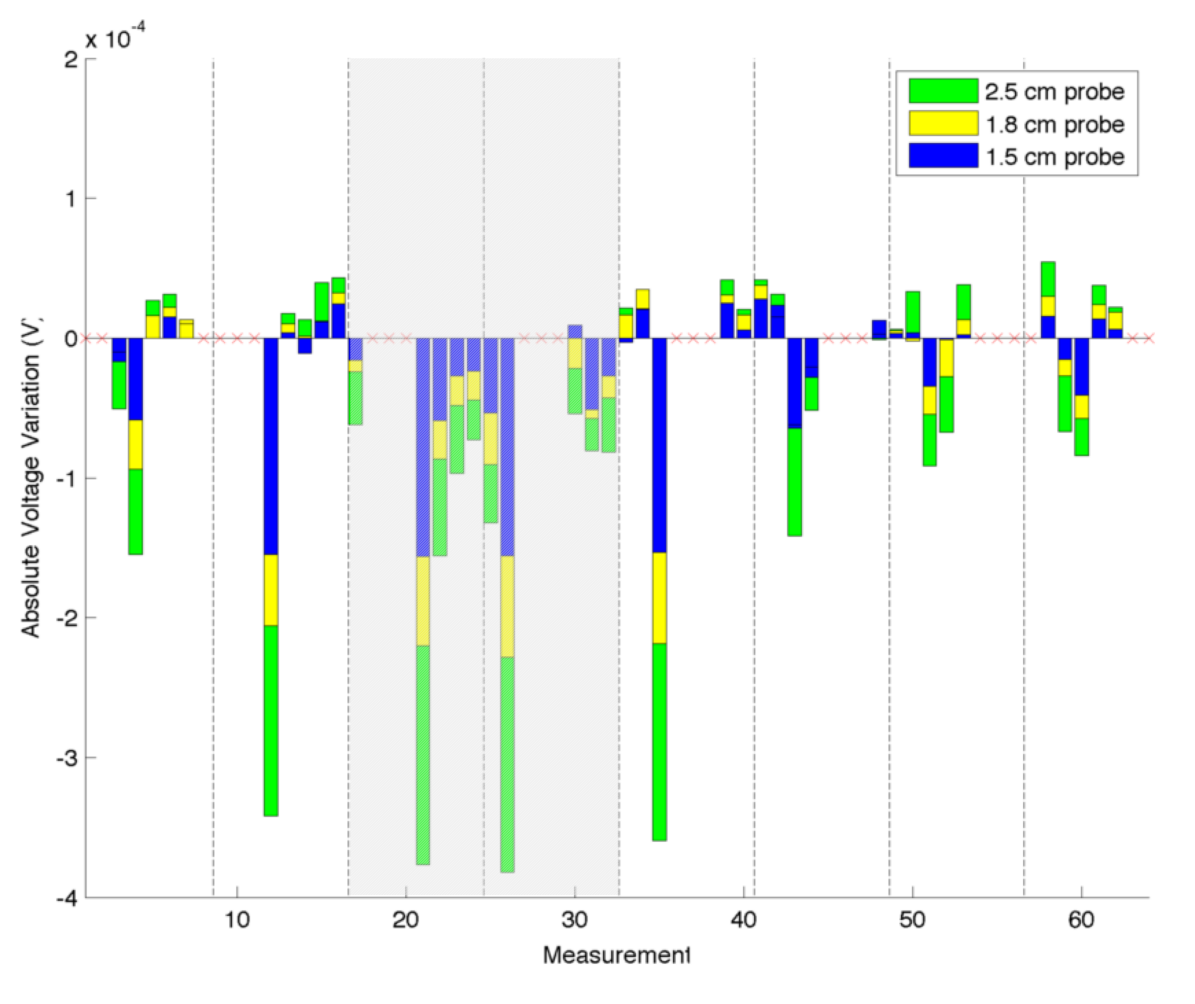
Difference in electric potentials measured when different probes are placed in the region facing Electrode 4 (E4). The probes have different size, while the applied force is kept constant. As in the previous image, the shaded regions correspond to E4 acting as driving electrode.

Considering the same electrode configuration, we evaluate the changes in the absolute voltage variation as a function of the probe size, while applying a constant force (5 N) over them. Three different circular probes with radius of 15 mm, 18 mm and 25 mm were placed independently in front of the region facing Electrode 4 (E4). Figure 10 shows the results of the experiments. As the graph suggests, with the increase of the probe size, the variations of the electric potential increase. This was expected, since a larger probe generates a wider inhomogeneity area, which is the cause of a consequent voltage drop. Even if the probe size is changed, it is still possible to notice a large negative change in the electric potentials when E4 is considered in the measure. Similar effects are expected in the case where the probe area remains constant and the applied force is increased. This consideration requires additional verification, which is left as future work.

### 4.3. Conductivity Map Reconstruction

**Figure 11:**
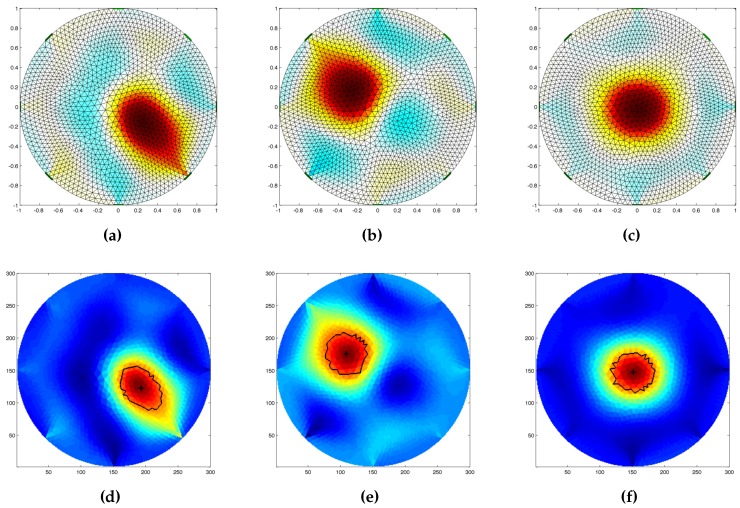
Reconstructed conductivity map of different stimuli applied over the artificial skin by a circular head probe of 18 mm radius. The probe was placed (**a**) between electrodes E3 and E4; (**b**) between electrodes E7 and E8; (**c**) in the centre of the sensing area. Figures (**d**–**f**) show the processed data, respectively, of figures (**a**–**c**). In each of the processed images, the centre and the computed boundary of the detected inhomogeneity are shown.

In this phase, we validate the capabilities of the inverse solver to reconstruct the pressure map of forces acting over the sensing area of the artificial skin. As previously introduced (Section 3), in order to guarantee the solvability of the inverse problem, we precondition the solution using NOSER (Newton’s one-step error reconstructor) Gauss–Newton normalised difference inverse. For the initial test, we used a single circular probe with a radius of 18 mm, and applied it in different regions of the sensing area. Figure 11 shows the results obtained though the reconstruction algorithm. It is worth noting that no filtering of the data was performed during any phase of the process. In the image, the inhomogeneity is clearly distinguishable from the background, but its shape is not well defined. As the images suggest, the closer the object is placed to the centre of the sensitive area, the interaction between the applied force and the constraint produced by the electrodes is reduced (Figure 11c). On the contrary, the closer the probe is placed toward the boundaries (and thus closer to the electrodes), the less precise is the reconstructed shape of the probe. This can be caused by the effect the pressure has over the applied point and the constraints due to the presence of the electrodes. To partially overcome this issue, we processed the reconstructed map by thresholding it and considering only the pixels having value up to 70% of the detected maximum value. Figure 11d–f shows the reconstructed conductivity map with its centre superimposed.

**Figure 12:**
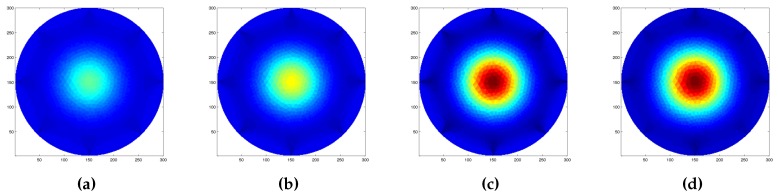
Reconstructed image as a function of different probes applied at the centre of the artificial skin. The results provided are, respectively, for: (**a**) a circular probe with 15 mm radius; (**b**) a circular probe with 18 mm radius; (**c**) a circular probe with 25 mm radius; and (**d**) a square probe with with a 15 mm side. Due to the low resolution of the system, it was not possible to clearly discriminate between the different shapes. Nevertheless, the change in the probe size can be easily identified.

We also tested the inverse solver capabilities to distinguish between different probe shapes when applied independently over the same position. In order to have less interaction with the boundaries, that have proven to suffer from a highest distortion due to the interaction of the probe with the electrode, we decided to use the central region for the tests. Figure 12 shows the results of the reconstruction using the three circular probes, and a square one under the effect of a constant force of 3 N. Due to the intrinsic low spatial resolution of the EIT method in this region it was not possible to clearly discriminate between the sharp-edged probe (Figure 12d) from the rounded-edged ones. Nevertheless, the change in the probe size (Figure 12a–c) can be clearly identified as the increase of the detected maximum value when a common color scale is used. 

### 4.4. Multi-Pressure Test

**Figure 13:**
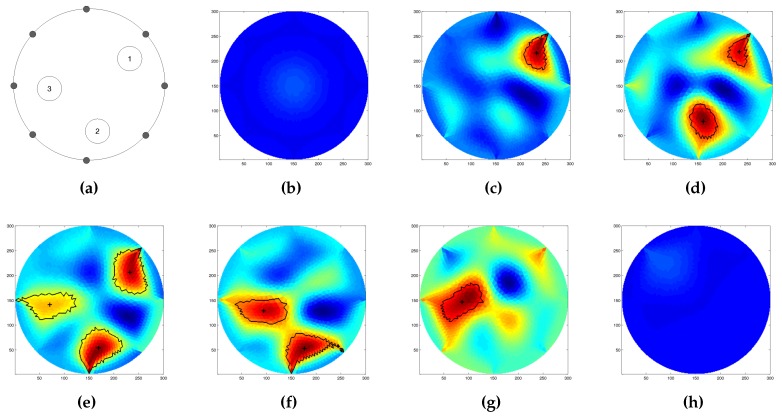
Selected frames in the multi-pressure experiment. (**a**) Probe positions; (**b**) reference voltage, (**c**–**e**) sequential load; and (**f**–**h**) unload of the different probes. In each frame, for each probe, its centroid and the computed boundaries are shown.

Following the experimental design described in the previous sections, we tested the capabilities of the artificial skin in the detection of multiple events that occur sequentially and simultaneously over the sensing area of the artificial skin. Three probes with the same size and shape (circular section with 15 mm radius) were manually placed and then sequentially removed over the sensitive surface of the artificial skin. In order to ensure equal applied force, a mass of 400 g was placed over each probe. As in the previous case, no signal processing was performed prior to or during the image reconstruction; the only operation that was performed was the precondition of the solver to ensure solvability. In Figure 13, selected frames obtained through the reconstruction process are presented.

As in the previous cases, we processed the conductivity map to obtain the area where each probe was acting. We first applied the same methodology as in the precious cases, but were unable to correctly identify the third probe when it was placed over the sensing area together with the other two (Figure 13e). For this reason, we reduced the threshold value from 70% of the maximum value to its 60% value. As the images suggest, the shapes of the probes could not be correctly detected, as a consequence of their position and the interaction of the electrodes In addition, the artificial skin showed some hysteresis after the removal of all the probes. This could be caused by the partial detachment of the conductive textile from its substrate, or by the change of the conductivity of the sensing layer as a consequence of the applied forces.

### 4.5. Application over Curved Surface

A last series of experiments was carried out to prove the adaptability of the artificial skin to conform to different geometries while maintaining its sensing property. Furthermore, it was also possible to test the efficiency in determining changes in shape (e.g., bending) due to the application of the artificial skin over different geometries, comparing the acquired electric potentials with the ones acquired over a flat surface. In this section, we will present the results obtained after the application of the artificial skin over different curved and deformable objects.

**Figure 14:**
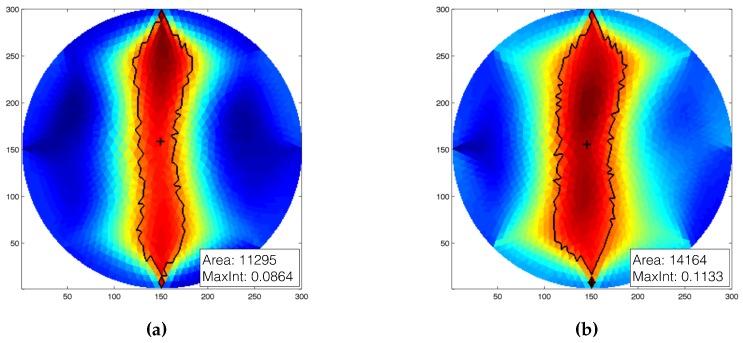
Results of the application of the artificial skin over a curved surface. The reconstructed conductivity map was generated by using as reference the measurements taken over a flat surface with no load acting over the artificial skin. In this experiment, we used two polyethylene foam extruded cylinders with diameters (**a**) 40 mm and (**b**) 70 mm. As the results suggest, as the diameter of the underlying object increases, the maximum intensity and the area of the detected inhomogeneity increase.

As an initial test, we placed the artificial skin over a flat surface and then took a series of electric potential measurements. With those values stored as reference, we placed the artificial skin over different cylindrical objects—two polyethylene foam extruded cylinders—with radii, respectively, of 40 mm and 70 mm. The two cylinders were then placed over a flat surface in order to provide sufficient support, and avoid their bending. Due to the presence of the foam substrate and the small size of the underlying object, the artificial skin could not conform perfectly to it. Thus, in order to fully cover the cylindrical object, we ket the two free sides of the artificial skin together by means of a plastic strip. In this configuration, we performed the electric potential measurements. Figure 14a,b shows, respectively, the results for the two different cylinders. The reconstructed conductivity maps presented in the two figures show a different behaviour compared to the one seen in the the previous experiments. In fact, instead of having a ellipsoidal region, this type of deformation produces a more elongated one. By comparing the images in Figure 14, it is possible to notice a slight change both in the maximum value detected and in the computed deformation area, obtained as in the previous cases by thresholding the reconstructed map. These observations can can be used as a clue to discriminate between different types of deformations applied to the artificial skin.

**Figure 15:**
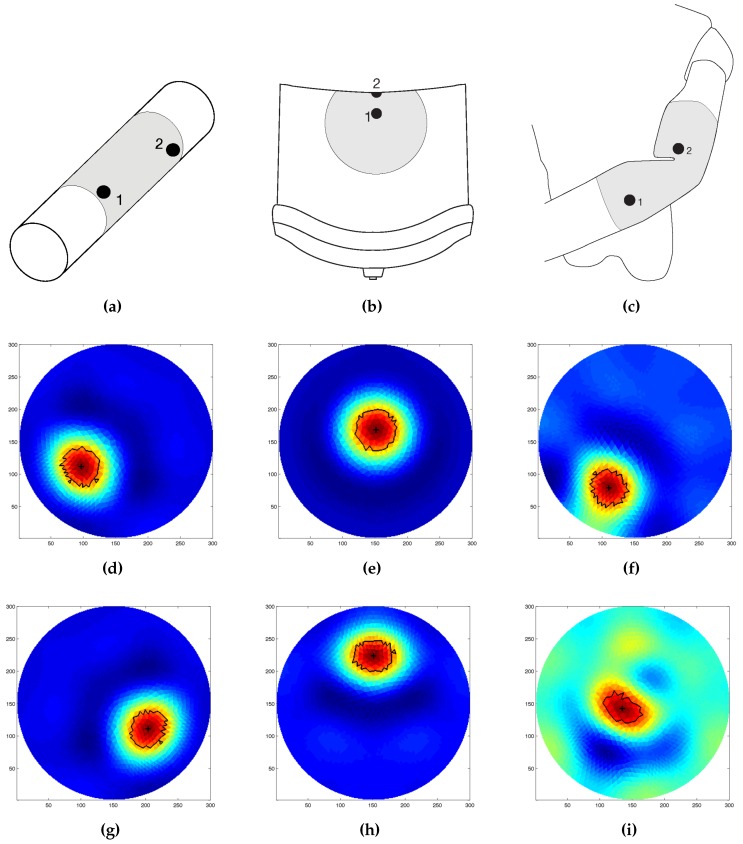
Example of conductivity map reconstruction over different geometries, results are grouped by column. First column (**a**,**d**,**g**) experimental setup and results when applied to a 70 mm radius cylinder (deformable). Second column (**b**,**e**,**h**) experimental setup and results when applied to the back of a chair (soft substate). Third column (**c**,**f**,**i**) experimental setup and results when applied to a mannequin arms with circumference of 30 mm (bendable). Results are ordered according to the number shown in their relative experiment setup. Last row of the Figure shows measurements performed when the probe was applied over the most curved area (**g**,**h**), and when the joint ange of the mannequin arm was changes (**i**). For each case, the results are obtained by applying a circular shape probe with 18 mm of radius.

Following the results obtained in the first part of the trials, we further tested the artificial skin’s ability to sense pressure when placed on curved geometries. We evaluated this property on the following objects: a 70 mm radius cylinder (deformable), the back of a chair (soft substate), and a mannequin’s arms with a circumference of 30 mm and a total length of 295 mm. Before proceeding with the measurements, we firmly attached the artificial skin to the underlying object in order to limit the error in the reconstruction due to changes in the electrode positioning. Where the object was not able to support itself (i.e., in the polyethylene foam extruded cylinder case), we placed the object on a flat surface for the duration of the test. We limited the experiments to a single probe of circular section and 18 mm radius. Differently from the precious case, reference measurements were taken after attaching the artificial skin on the curved surface. Results for each configuration are shown in Figure 15. As the images suggest, the artificial skin can provide similar results as the one shown in previous cases, even under large deformations.

## 5. Discussion

The main aim of this work was the investigation and development of a low-cost adaptable smart sensor that can adapt to different configurations while maintaining its sensing capabilities. Compared to currently available solutions, the system under study is smaller and simpler, but similarly effective. To prove the capabilities of the developed sensor, we tested the system under different configurations, the results of which we will describe in this section.

A first series of experiments was performed to characterise the behaviour of the artificial skin. Concerning the specific results obtained in the indentation tests (Figure 8, Figure 9 and Figure 10), when compared to reference measurements (Figure 5 and Figure 6), it is possible to notice a common pattern of signal variation as a consequence of the presence of a probe (in this specific case, a 15 mm circular section one) that applies a force in the region directly facing one of the electrodes under test. The graphs shown in the figures depict the absolute variation of the electric potential with respect to a reference voltage ($V_{meas}-V_{0}$). In these, the presence of the inhomogeneity can be noticed as a drop in the electric potentials measured at the electrode under test—i.e., E2 in Figure 8 and E7 in Figure 9. The variation is caused by the change in conductivity of the conductive layer used for sensing as a consequence of the normal force applied over it. As Figure 10 suggests, the change of the electric potentials is directly related to the size of probe used to apply a constant force.

Following the obtained results, we move toward the evaluation of the conductivity map reconstruction algorithm. To prove its effectiveness, we test the system under different configurations, in which we applied different probes independently or simultaneously over the artificial skin. Concerning such experiments, we refer to Figure 11, Figure 12 and Figure 13. The reconstructed pressure map (which is equivalent to the conductive map of the domain under study) was obtained by solving the associated inverse problem using a dynamic imaging approach. This consists of comparing a reference reading taken when no load was applied over the surface with a later one where a force was applied. Since the EIT inverse problem is ill-posed and ill-conditioned, in order to ensure uniqueness of the solution, it is needed to regularise the model during the inverse problem solution Parameters to correctly tune the solver were selected after a series of trial and error experiments, and have been proved to be appropriate by evaluating them under different system configurations. It is worth noting that we kept the parameters constant in all experiments. Because of the low number of emitters and detectors used to build the electronic skin tested in this work, we did not expect a good spatial resolution for the contacts applied over the sensing area. Despite this, results provided in Figure 11 show that the inverse solver can correctly identify the position of the applied force, but could not clearly identify its shape. To have a sharper identification of such area, we further process the data by thresholding it, identifying the pixels having value up to 70% of the maximum value present in the map. Considering the limitations due to the low resolution, we further test the system capabilities to discriminate between different probe sizes and shapes. The results of these experiments are provided in Figure 12. As the reconstructed images suggest, the system is not able to correctly discriminate between different probe shapes (i.e., circular and square head), but it is able to correctly capture the differences in size (i.e., 15 mm, 18 mm, 25 mm for the circular probe, and 15 mm side for the square-headed probe). As a last test for this series of experiments, we evaluated the capability of the system to determine the presence and position of multiple probes that act sequentially and simultaneously over the sensing area of the artificial skin. For this series of experiments, we used three identical probes (circular section with radius of 15 mm) that were loaded and unloaded in the same order. Figure 13 shows selected frames of the process. In the figure, it is possible to clearly distinguish the area where the probes act, but due to the low spatial resolution, it is not possible to correctly identify their shapes. In addition, when all the probes were placed over the surface, in order to detect their positions, we had to change the value of the thresholding parameter used in the image segmentation. The use of a larger number of components, along with the introduction of more advanced coupling schemes for emitter–detector pairs can in the future improve the spatial performance and thus overcome some of the problems that the system is currently facing.

We further tested the capabilities of the artificial skin by evaluating its ability to adapt to different geometries while maintaining its sensing performance. Figure 14 and Figure 15 show the results related to this last series of experiments. In the first phase, we tested the system’s ability to detect a change in the underlying geometry caused by a significant change in the potential measurements (Figure 14). We first acquired a series of electric potentials with the artificial skin placed over a flat surface, and then manually placed it over a curved one. As curved surfaces, we used two different cylindrical objects with radii 40 mm and 70 mm, respectively. As the results indicate, when compared to the reconstructed images from the flat cases, the reconstructed images show a different “shape”. These results, together with the fact that the maximum detected value and the size of the “deformation area” increased with respect to the radius of the underlaying cylinder, can be used as a clue to discriminate between different events (i.e., bending vs. touch) and their intensity (e.g., bending angles). Additionally, we tested the sensing capabilities of the system to provide reliable pressure readings when subjected to large deformations. In order to prove this, we tightly attached the artificial skin to objects having different shapes, and then performed pressure sensing. Contrary to the precious case, we acquired both the reference measurements and the later ones when the artificial skin was already placed over each object. Doing so, all of the deformations that occur as a consequence of the underlying geometries are negligible, since they are already taken into consideration in the reference measurements. Figure 15 shows the results for each of the considered scenarios. As the results suggest, the system maintains its sensing capabilities and can identify the position where the probe was applied. Some issues were noticed when the sensor was applied over the mannequin, especially in the joint area. If the joint is moved after acquiring the initial reference measurements (Figure 15i), the measurements acquired later are affected by a planar force applied by the joint—i.e., the joint stretching the material. The sensor can be still used in this situation by taking new reference measurements according to the information provided by the joint encoder. For all the other cases, results remain valid, even when the probe was applied over the most bended area (Figure 15g,h).

## 6. Conclusions

In this paper we presented an artificial skin able to easily conform and adapt to different shapes while keeping all of its sensing capabilities. It is easy to fabricate, requires low power consumption, and allows continuous and distributed sensing over the entire surface, since it does not require discretised components or connection wires within the sensing area. This was made possible by exploiting as working principle a tomographic technique known as Electrical Impedance Tomography (EIT). The technique allows inference of the structure of the studied media by injecting an electrical current into it and by measuring electric potentials from its boundaries. The use of this technique allows continuous distributed sensing without the need of active components directly embedded inside or beneath the sensing area. A direct consequence of the use of this approach is the simplification of the fabrication process (the sensing area can be any conductive material) and a smooth extension to higher resolution. In addition, not having connecting cables that sit in the sensing area allows the developed of smart skins that can have a wider surface and that can easily adapt to different substrates and shapes. These are features that are not always achievable with the classical transduction methods. Moreover, an important aspect is the one related to the architecture used in the system. In fact, in order to increase the spatial resolution, the number of active components increases linearly with the length of the sensing area boundary, rather than with its area. This can be proven to be advantageous in terms of both costs and power consumption. On the contrary, the increase of active components places a larger burden in terms of computation time for the voltage acquisition system. Similar approaches have already been presented, but our sensor structure presents additional system simplifications and hardware modularity that allows it to be more easily applied in situations where not only the sensor should adapt to different geometries, but also the underlying structure can change its shape.

In order to prove the feasibility of the system, we tested it under different situations by varying the size, shape, and the number of the probes acting over the artificial skin surface. Although we were not able to correctly distinguish between the different probe shapes, we managed to detect their positions in space and have some idea of their size. Furthermore, we tested the system in configurations where it was subjected to large deformations and applied over different geometries. In every case, the system performances were more than acceptable. Despite the positive results, the artificial skin has some limitations, especially related to the spatial resolution and the detection of small forces. It is possible to partially overcome the issues by increasing the number of electrodes used, or by changing the current injection and voltage acquisition pattern, with the drawback of the increasing the acquisition time and increasing the computational cost of the solution of the inverse problem.

Future works include the development of a larger electronic skin with higher density of active components, while keeping the focus on fast processing for real-time applications by exploring different coupling schemes. Additional work should also be done on the hardware side, by adding a signal conditioning stage to ensure more precise measurements. Another method to increase the system performance is to couple the current sensing methodology with capacitive measurements. This method has proved [32] to performs better than resistive both in detecting conductive an non-conductive objects. Since this sensor offers properties needed in the domain of soft robotics, we are currently investigating novel materials to be used as conductive layers that have similar properties to the one used in this work. In fact, the use of textiles is not suitable, since it is not possible to firmly attach them onto the material used to build such devices (i.e., silicon rubber).
